# Introducing a Framework for Surgical Discharge Summaries to Improve Accuracy and Succinctness in a Busy District General Hospital

**DOI:** 10.7759/cureus.71789

**Published:** 2024-10-18

**Authors:** Eesaa Longden, Aashna Sikka, Ashwin Bobby, Ravi Goel, Mariam Dean

**Affiliations:** 1 Psychiatry, Royal Blackburn Hospital, Blackburn, GBR; 2 Emergency Medicine, Royal Blackburn Hospital, Blackburn, GBR; 3 Surgery, Royal Blackburn Hospital, Blackburn, GBR; 4 Vascular Surgery, Royal Blackburn Hospital, Blackburn, GBR; 5 Urology, Royal Albert Edward Infirmary, Wigan, GBR

**Keywords:** continuity of patient care, discharge summary, electronic patient records, postgraduate education, quality improvement projects, surgical notes

## Abstract

Background

Discharge summaries (TTOs) are essential documents in the effective communication between primary and secondary care, particularly in conveying critical post-discharge instructions to patients. Inconsistencies and omissions in TTOs can significantly undermine patient outcomes and disrupt continuity of care. This is particularly relevant to surgical patients, who often require specific follow-up care such as the removal of clips or drains shortly after discharge. Following the recent transition from paper-based to electronic records at a busy district general hospital (Royal Blackburn Hospital, Blackburn), the quality of TTOs was noted to be substandard. This quality improvement project aimed to enhance the accuracy and clarity of surgical TTOs.

Methods

A targeted framework was developed in collaboration with local consultants and the consideration of national guidelines to guide the content of surgical TTOs, focusing on five essential components: Reason for Admission, Intervention, Surgical Details, Discharge Plan, and Follow-up Instructions. Initial retrospective data analysis included TTOs (n=60) across five surgical wards, evaluating their quality against the framework. The framework was then introduced via educational initiatives and integrated into the hospital’s electronic patient record (EPR) system (CERNER). The impact of these interventions was assessed through data collection after two Plan-Do-Study-Act (PDSA) cycles.

Results

Baseline data highlighted significant deficiencies; 42 (70%) of TTOs were missing at least one key element, with missing follow-up details most often the reason. Many TTOs also included excessive or inappropriate information, and their format varied greatly depending on individual writing styles. Following the first PDSA cycle and the introduction of the framework, the number of TTOs containing all essential data points increased by 14 (30% increase), while those missing two or more elements decreased by 16 (48% decrease). After the second cycle, further improvements were observed, with the number of TTOs missing one or more data points decreased by 6 (21%). Despite the overall progress, follow-up information continued to be the most frequently omitted element. Feedback from resident doctors was positive and the unanimous opinion was that the framework improved not only the quality of TTOs, but also how efficiently they were written.

Conclusions

Implementing a standardised framework significantly improved the quality of surgical TTOs, particularly by increasing the inclusion of critical information. These results are encouraging. However, anecdotal evidence suggests there is a lack of training in writing TTOs at both undergraduate and foundation levels. Ongoing efforts are required to address these areas to ensure a sustained improvement in quality.

## Introduction

Discharge summaries (sometimes referred to as TTOs) are crucial documents in healthcare. Not only do they form the foundation of communication between primary and secondary care facilities, but they also play a crucial role in educating patients about their admissions to the hospital. These summaries provide patients with vital instructions upon their discharge such as medication continuation, follow-up appointments, and even prognosis [1]. There has been clear evidence demonstrated in other studies that prove high-quality TTOs are directly linked to improved patient outcomes [2]. 

Due to their dualistic nature as a document that should convey information appropriately to both specialists and laypersons, they can be tricky to get right. Additionally, in an era defined by a struggling National Health Service with high burnout rates and staff shortages [3], finding the time to compose a well-thought-out TTO can be difficult. Nevertheless, as a common task often delegated to the most junior doctors, it is essential that these individuals possess both the confidence and competence to complete them accurately and without significant omissions. 

Unfortunately, due to a lack of guidance and a constantly rotating junior workforce, the inconsistency and inadequacy of TTOs in the surgical setting at the hospital in question quickly became apparent. Compounded by the trust-wide transition from paper-based to electronic records only eight months prior to this project, a clear standard needed to be outlined. 

Studies have shown that a reduced workload on doctors can significantly improve TTO quality [4]. By designing, implementing, and improving a clear framework for TTO contents, this quality improvement project (QIP) aimed to reproduce these results and improve doctor confidence overall in this area. 

This QIP was previously presented as an oral presentation at the 2024 SUPTA National Surgical Conference on August 24, 2024, and as a poster presentation at the UKDHC Annual Scientific Informatics Conference on September 4, 2024.

## Materials and methods

To best synthesise a TTO framework that would include relevant and essential elements, local surgical consultants were asked for their advice and existing guidelines from the Royal College of Physicians (RCP) and the Royal College of Surgeons (RCS) were considered [5]. There was a consensus that intervention would be welcome and after some deliberation, a simple framework was agreed upon. The five fundamental subheadings of the framework were: Reason for Admission, Intervention(s), Surgery: If so, what and when, Plan on discharge and Follow-up: If so, when and with whom. 

This framework aimed to guide the writing of efficient, succinct TTOs that include vital information for both patients and doctors. The first PDSA cycle (Plan, Do, Study, Act) of the project involved raising awareness of the framework in teaching sessions and placing posters of the framework in surgical ward offices. The teaching sessions were formally organised and delivered to groups of resident doctors working in surgery. They focused on two areas: succinctly displaying exemplary TTOs and comparing them with some in need of improvement and raising awareness of the new framework and where to find it. After the first cycle, a questionnaire was distributed among resident doctors to gain feedback on the implementation of the framework in clinical practice. Their responses helped to guide the second cycle which involved integration of the framework into the hospital's Electronic Patient Record (EPR) system in the form of an e-template. 

Data collection 

Initial data were gathered retrospectively from records of admissions and discharges across five surgical wards to identify recently completed TTOs. Patient-identifying numbers were chosen at random from a timeframe spanning one month. 

A total of 60 TTOs were selected using random number generators analysed across these wards and evaluated in a binary fashion against the established criteria points laid out in the framework above. These included 20 from the Emergency Surgical Unit and 10 from each of the other wards. Some qualitative observations were also made to account for certain issues that were not covered in the set criteria, such as excessive or unclear information. 

After both PDSA cycles, data was re-collected in the exact same way as before, with the same reviewers, to reduce the chance of discrepancies or bias.

## Results

Initial results 

The results of the preliminary round of data collection highlighted the deficiencies in TTOs, proving that intervention was required. Missed points were consistent across all wards, emphasising the existence of a systemic, rather than an isolated problem. 

**Figure 1:**
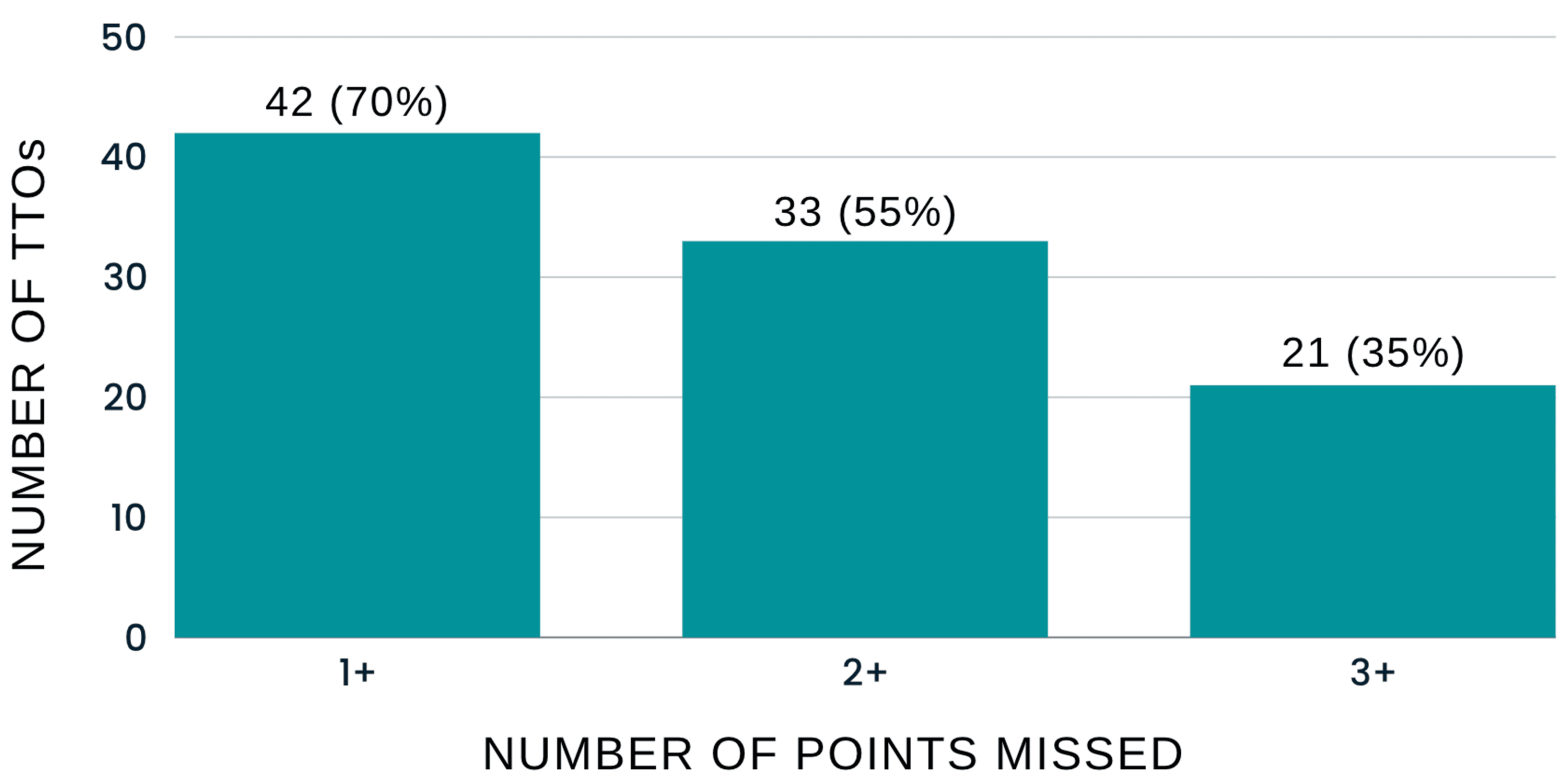
Relationship Between the Number of TTOs and Points Missed (Baseline Data) TTOs: Discharge summaries

**Figure 2:**
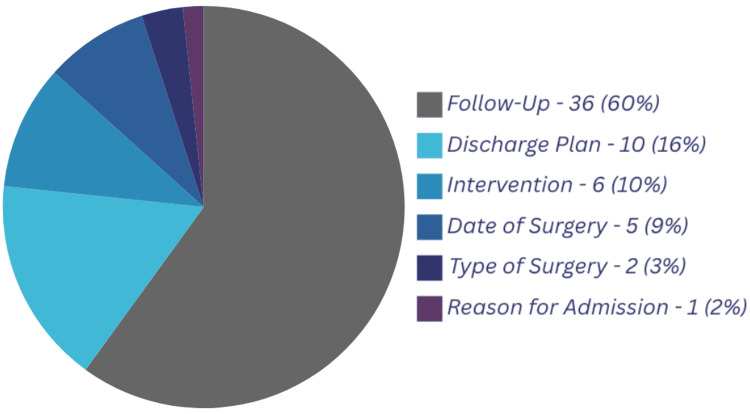
Percentage Breakdown of Missed Points in TTOs (Baseline Data) TTOs: Discharge summaries

Of the TTOs analyzed, 42 (70%) were found to be missing at least one of the essential points outlined in the framework, 33 (55%) missed two or more points and 21 (35%) missed three or more points (Figures 1, 2).

First cycle 

**Figure 3:**
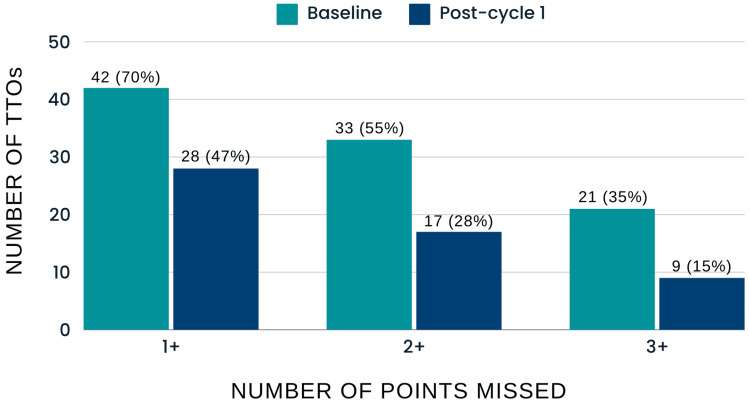
Comparison of Number of TTOs and Missed Points Pre- and Post-PDSA Cycle 1 TTOs: Discharge summaries; PDSA: Plan-Do-Study-Act

One month after the introduction of the framework, data was re-collected, revealing notable improvements. The number of TTOs containing all relevant data points increased by 14 (30% increase), while the number of TTOs missing at least one point decreased by 14 (33% decrease). Additionally, the number of TTOs missing two or more points was 17 (48% decrease), with only nine (15%) of the TTOs missing three or more points, a significant improvement from the pre-intervention figure of 21 (35%). However, of the total 44 missed points this cycle, 40 (90.9%) were related to follow-up information, up from 60 out of 100 (60%) missed total points in the preliminary data, indicating that while the framework improved overall compliance, the inclusion of follow-up details remained insufficient (Figure 3).

Second cycle 

**Figure 4:**
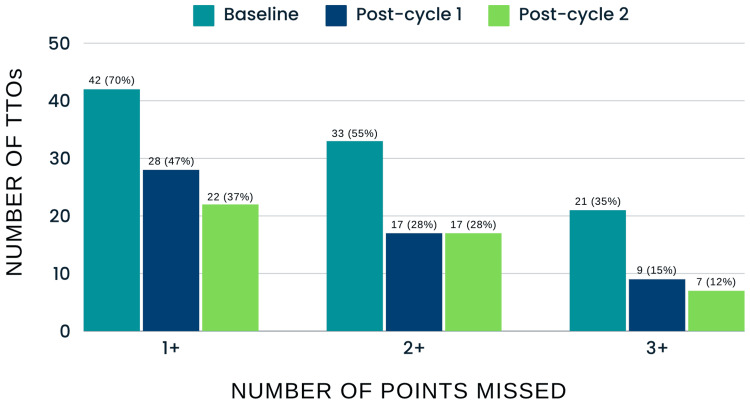
Comparison of Number of TTOs and Missed points between baseline data, post-PDSA cycle 1 and post-PDSA cycle 2 TTOs: Discharge summaries; PDSA: Plan-Do-Study-Act

Based on feedback from the surgical staff, the most common suggestion was to implement the template directly into the hospital's EPR system (CERNER) to improve efficiency and serve as a reminder to include follow-up details. In response, an auto-text shortcut was created and distributed to staff across the wards. After one month, data was re-collected, showing slight improvements in most areas. The number of TTOs missing one or more data points decreased by 6 (21% decrease). However, the number of TTOs missing two or more points remained at exactly 17 (28.6%), likely due to summaries previously missing three or more points improving slightly and falling into this category. Furthermore, the total number of points missed across all TTOs increased slightly from 44 to 48 (9.1%), although this change was not significant. Follow-up details remained a significant issue, with 36 out of 48 (75%) of missed points attributed to missing follow-up information, a slight improvement from 90.9%. Additionally, 22 (37%) of TTOs were noted as being overly detailed, containing excessive information (Figure 4).

Qualitative results 

Qualitatively, three key issues were identified. 

First was the incorrect use of outdated plans as discharge plans. A recurring theme was the copy-paste of post-operative, ward round and even admission plans into the TTO. This led to confusing discharge plans, very often with no mention of follow-up at all. 

Second was the duplication of admission clerking notes into the TTO. The EPR system used to generate TTOs (CERNER) has specific formatting requirements. There are separate text boxes for the clinical summary and plan. Due to inconsistent use of both boxes, often by multiple clinical teams, they can be populated by incongruous information. Without thorough editing, this conflicting information can end up in the final TTO. 

Third, some TTOs were noted to have excessive information. These contained substantial amounts of information, sometimes including entire scan results, multiple blood test results and even input from other members of the hospital's multi-disciplinary team. Whilst this is important to consider, incorporating such information into every TTO would be not only be time-consuming and difficult for resident doctors to compose, but also for General Practice staff to read and patients to understand. 

## Discussion

There are many barriers to resident doctors completing TTOs to a high standard in surgical wards. Patient turnover occurs at a higher rate than in the rest of the hospital, especially in the Emergency Surgical Unit wherein TTOs lack the most important information. This is an issue compounded by the mandated and frequent rotation of resident doctors who never get to know their patients as well as they should. 

Despite the existence of RCP and RCS guidelines, resident doctors can struggle to effectively summarize the relevant details of a patient’s stay in the hospital. Anecdotal evidence suggests a lack of formal training in the creation of TTOs at both undergraduate and resident levels. This re-enforces a potentially unaddressed gap in training and implies a lack of awareness of national guidelines. 

Similar studies and QIPs have reinforced these findings, providing further evidence of an issue prevalent across the National Health Service (NHS)[6]. 

Limitations 

The project had several limitations. First, there is a degree of subjectivity in determining whether TTOs contain excessive or irrelevant information, which may have affected the consistency of data collected by different members of the QIP team. Second, the criteria developed by the team were specifically tailored to surgical patients and may not apply to outliers from non-surgical specialties who were also treated on the wards. This limits the broader applicability of the framework. Third, despite multiple targeted efforts to raise awareness of the framework, some staff remained unaware and did not utilize it, as evidenced by gaps in compliance across various rounds of data collection. Finally, the main area that this QIP failed to adequately address was the lack of follow-up information included in TTOs. Whilst there was a small improvement over the two cycles, the root causes of the issue were not tackled. These include a lack of senior-to-junior communication and a lack of specialty-specific knowledge around appropriate follow-up timescales among junior doctors completing TTOs. 

Future challenges

Future challenges for this project include increasing and maintaining awareness of the framework among staff, particularly as new resident doctors rotate through the wards. A sustained focus is needed to address why many staff continue to omit follow-up information in TTOs. Encouraging senior staff to consistently communicate follow-up plans at every opportunity could help improve this area. Educating more junior staff about how common presentations are followed up would also improve autonomy and confidence of doctors completing TTOs. Additionally, there is a need to address gaps in the UK medical school curricula regarding formal training on composing effective patient documentation, ensuring that junior doctors are better prepared for this crucial task.

## Conclusions

A two-cycle QIP was undertaken over a period of six months which focused on considerably improving the quality and succinctness of TTOs for surgical patients. A framework covering relevant information points was synthesised and approved by surgical consultants. Over two PDSA cycles, awareness was raised of the new framework, and it was implemented into the local EPR system. 

Significant improvements were observed in the quality of surgical TTOs over both cycles; however, several root causes were identified, and each should be addressed separately moving forward. Importantly, focusing on educational initiatives at the undergraduate and foundation level will be crucial in ensuring sustained high standards. 
